# Case Report: Enhanced Diazepam Elimination With the Molecular Adsorbents Recirculating System (MARS) in Severe Autointoxication: A Survival Case Report

**DOI:** 10.3389/fmed.2021.633250

**Published:** 2021-03-09

**Authors:** Anna Dobisova, Peter Vavrinec, Diana Vavrincova-Yaghi, Andrea Gebhardtova, Robert H. Henning, Aktham Yaghi

**Affiliations:** ^1^Faculty of Medicine, University Hospital Bratislava, Nemocnica Ruzinov, ICU, KAIM, Clinic of Anesthesiology and Intensive Care Medicine, Comenius University in Bratislava, Bratislava, Slovakia; ^2^Department of Pharmacology and Toxicology, Faculty of Pharmacy, Comenius University in Bratislava, Bratislava, Slovakia; ^3^Department of Clinical Pharmacy and Pharmacology, University of Groningen, University Medical Center Groningen, Groningen, Netherlands

**Keywords:** diazepam, mars, pharmacokinetics, elimination, autointoxication

## Abstract

**Objective:** Due to the extensive use of diazepam worldwide, self-induced intoxication is very common, yet rarely fatal. Nevertheless, the management of intoxication caused by extremely high doses of diazepam is not known, as well as the effectiveness of flumazenil, a specific benzodiazepine (BDZ) antagonist. Here we present the first report on the enhanced elimination (clearance) of diazepam using the Molecular Adsorbents Recirculating System (MARS) following autointoxication with an extremely high dose as part of a suicide attempt.

**Case:** A 44-year-old male patient was admitted to the ICU because of impaired consciousness following the ingestion of 20 g of diazepam. Blood and urine samples revealed high benzodiazepine levels. Repeated doses of flumazenil were without effect on consciousness. Following deterioration of the patient's clinical condition, including unconsciousness, hypoventilation, and decreased SpO2 (88%), the patient was intubated and mechanically ventilated. On the fourth day after admission, the patient was unresponsive, with no attempt to breath spontaneously. The plasma level of benzodiazepines was 1,772 μg/l. The elimination of benzodiazepines by MARS was attempted, continuing for 5 days, with one session per day. Five sessions of MARS effectively enhanced benzodiazepine elimination. After the first MARS treatment, the plasma level of benzodiazepines dropped from 1,772 to 780 μg/l. After the final MARS treatment on the eighth day, the patient was weaned from mechanical ventilation and extubated. Two days later, the patient was discharged to the internal medicine department and subsequently to the psychiatry department.

**Conclusions:** To the best of our knowledge, this is the first case reporting successful treatment of diazepam intoxication using MARS. In severe cases of diazepam intoxication, with prolonged unconsciousness and the necessity of mechanical ventilation, we suggest considering the use of MARS elimination therapy together with the monitoring of the BDZ plasma level.

## Introduction

Because of the extensive use of diazepam worldwide, self-induced intoxication is highly prevalent (1, 2). While common, single compound benzodiazepine (BDZ) intoxication rarely produces significant morbidity or mortality, the management of intoxication caused by extremely high doses of diazepam is not known, as well as the effectiveness of flumazenil, a specific BDZ antagonist. Because diazepam has a relatively long half-life (20–50 h), contains active metabolites (with a half-life of 36–200 h for the main active metabolite nordiazepam) and is highly bound to proteins (98%), the preferred strategy would encompass removal of the compound from circulation. Consequently, measures to enhance diazepam clearance may significantly shorten the duration of intoxication. The Molecular Adsorbents Recirculating System (MARS) is a removal strategy based on the principle of albumin dialysis and may therefore be especially useful in enhancing the clearance of (protein-bound) diazepam; however, its effectiveness in enhancing diazepam clearance following severe intoxication has not yet been explored. Here, we demonstrate for first time the effective enhancement of diazepam clearance by MARS, as used in a unique case of autointoxication featuring an extreme overdose of diazepam.

## Case

A 44-year-old male was admitted to the ICU because of impaired consciousness, with a Glasgow Coma Score of 11 (GCS: E4, M5, V2), after reported ingestion of diazepam. The intoxication occurred at the pharmacy during the evening hours. The patient was found unconsciousness by an employee, next to an emptied pharmaceutical powder canister that had contained diazepam.

Attempted suicide was suspected because of a letter left by the patient. Circumstances thus indicated that the patient had ingested 2,000 mg (20 g; 250 mg/kg) of diazepam, some 2–4 h prior to presentation at the ICU. The patient has no relevant medical, family, or psychosocial history, as well as no past interventions. No medical report about depressive disorder or previous suicide attempts exists. The patient worked as an economist in the pharmacy. On ICU admission, the patient's blood pressure was 110/70 mmHg, heart rate 90/min, with regular rhythm. The SpO2 was 90%, and the patient was hypothermic (35.5°C). The patient's weight was 80 kg, height 175 cm, and BMI 26.12 kg/m^2^. The patient had no renal or liver dysfunction. Plasma analyses revealed no changes in urea (3.27 mmol/L), creatinine (52.9 μmol/L), bilirubin (6.39 μmol/L), AST (0.37 ucat/l), ALT (0.21 ucat/l), and AMS (1.38 ucat/l). Further physical examination revealed no abnormalities. Blood and urine samples were taken for toxicology investigation. According to protocol, the patient was administered 75 g of activated charcoal and 20 g of MgSO_4_ by nasogastric tube every 6 h. I.v. flumazenil (0.5 mg) administration was without effect. Because of agitation, intermittent i.v. administration of propofol (50 mg) was used. BDZ intoxication was confirmed from blood and urine samples 5 h after admission, with plasma concentration above the upper detection limit of 2,000 μg/l. Plasma and urine BDZs were measured using DRI® Benzodiazepine Serum Tox Assay and Emit® II Plus Benzodiazepine Assay for urine. Because of hypoventilation and irregular breathing, decreased SpO2 (to 88%) and impaired GCS (to 3), the patient was intubated and mechanically ventilated starting from 5 h after admission without the need for additional sedation. Subsequent repetitive administration of flumazenil (0.5 mg i.v.; three times) was without effect.

During the subsequent days, the patient remained unconsciousness with an absence of spontaneous breathing. Plasma BDZ remained above the upper detection limit, which prompted the start of MARS on the fourth day of admission and its continuation for 5 days, one session per day. Informed consent was obtained from the patient's relatives.

**Figure 1:**
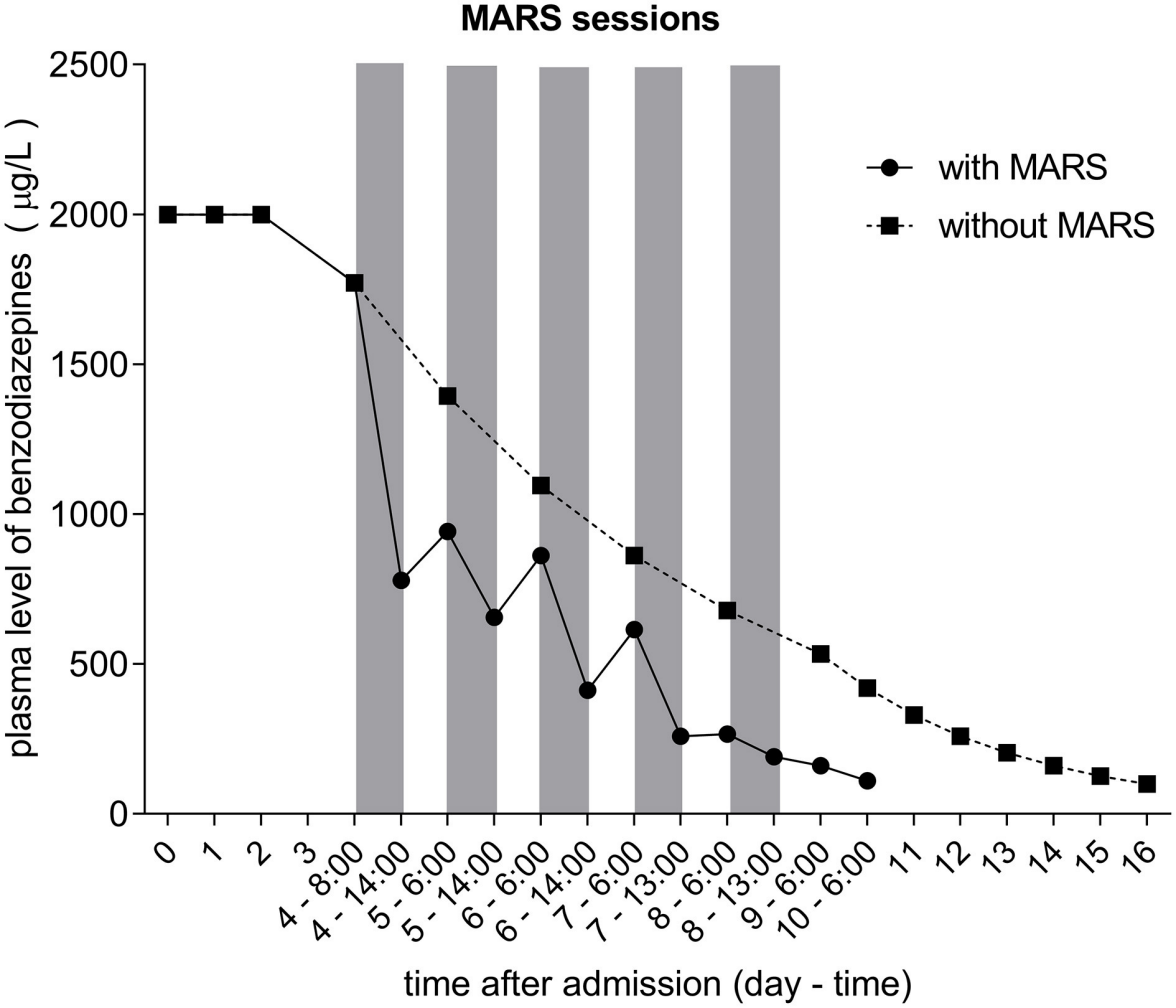
Plasma level of benzodiazepines throughout the study expressed as μg/L. The five MARS sessions are indicated by gray vertical bars. Dotted curve represents estimated plasma level of benzodiazepines calculated using t1/2 of 52 h. Total plasma benzodiazepines were assessed by the DRI® Benzodiazepine Serum Tox Assay.

Throughout the course of MARS, plasma levels of BDZ were measured twice daily—at 6:00 (before the MARS session) and at around 14:00 (after the MARS session). The first MARS session lowered the plasma level of BDZ from 1,772 to 780 μg/L, and subsequent sessions further lowered the BDZ levels (Figure 1, Table 1). By day eight, the plasma level of BDZ had dropped to 191.2 μg/L. The patient regained consciousness and was weaned from mechanical ventilation and extubated. Two days later, the patient was discharged to the department of internal medicine and subsequently to the department of psychiatry.

**Table 1:** Benzodiazepines plasma levels before and after the MARS sessions.

**Day**	**BDZ plasma before MARS (μg/L)**	**BDZ plasma after MARS (μg/L)**	**Decrease by MARS (%)**	**Increase after MARS (%)**	**Absolute increase after MARS (μg/L)**	**Estimated removal (mg)**
4	1,772	780	56			119
5	943	656	30	21	163	34
6	862	413	52	31	206	29
7	616	259	58	49	203	43
8	267	191	28	3	8	9
9	161	112	30	−16	−30	

*The effectiveness of MARS is expressed as the percent of plasma level decrease and tissue release expressed as the percent increase after mars. The absolute increase after MARS and estimated removal by MARS were calculated from volume of distribution of 1.5 L/kg, body weight of 80 kg. Total plasma benzodiazepines were assessed by the DRI® Benzodiazepine Serum Tox Assay. Total plasma benzodiazepines were assessed by the DRI® Benzodiazepine Serum Tox Assay*.

As a result of MARS, plasma BDZ levels were effectively lowered by a similar percentage at each session, averaging 45% (Figure 1, Table 1). In contrast, changes in the plasma BDZ level following each MARS session showed a different pattern, with a substantial and increasing percentage of nightly rebound following the initial three MARS sessions, followed by an abrupt halting of the increase, and even a net removal of BDZ following the last MARS session (Table 1). Of note, when expressed as ug/L change, the nightly rebound following the first three MARS sessions was similar.

Plasma BDZ levels during the last measurements, when MARS had been stopped, allowed calculation of the elimination half-life (t1/2; assuming a volume of distribution 1.5 L/kg and a body weight of 80 kg). During the first time interval, the BDZ level dropped from 191 to 161 μg/l in 17 h; consequently the calculated elimination t1/2 was 69 h. During the last 24 h of measurements, the BDZ level dropped from 161 to 111 μg/l; therefore, calculated t1/2 was 45 h. Overall, calculation of the elimination half-life over the last three time points, when the plasma BDZ level dropped from 191 to 111 μg/l in 41 hours, rendered a similar result, with a t1/2 of 52 h.

## Discussion

MARS is a system of extracorporeal albumin dialysis that is mostly used in patients with established liver damage. The concept of albumin dialysis depends on the elimination of protein-bound molecules and can also be used with intoxication in order to remove toxicants. To the best of our knowledge, this is the first case reporting accelerated elimination of diazepam using MARS.

Given the excessive dose of diazepam taken by the patient, a poor prognosis was foreseen. Because of reported high diazepam albumin binding (98%) (3, 4), it was hypothesized that albumin dialysis would enhance the clearance of the plasma protein-bound diazepam. However, both *in-vivo* and *in-vitro* evidence on the impact of MARS on BDZ elimination is scarce. Specifically, one *in-vitro* study focuses on the impact of albumin stabilizers (5) and another on the impact of the dialysate albumin concentration on removal efficacy (6), rather than on the impact of MARS on diazepam elimination itself. Thus far, one porcine *in-vivo* study reported MARS to have efficiently removed midazolam in the setting of induced acute liver failure (7). This experimental *in-vivo* study supported our decision to use MARS to eliminate BDZs from the patient's body.

Our data show that MARS efficiently lowers plasma BDZ levels, as single sessions resulted in an average drop in plasma BDZ of 43%. However, we also observed a significant rebound of plasma BDZ levels following the initial MARS sessions, which may have been the result of accumulated drug recirculation from plasma protein binding sites or redistribution from peripheral compartments, such as adipose and muscle tissue. Interestingly, absolute plasma BDZ levels increased similarly after the three initial MARS sessions, resulting in an increasing percentage of nightly plasma level rebound. This likely represents a release of accumulated drug from peripheral compartments at zero-order kinetics. This novel finding not only reflects the release mechanism of diazepam from tissue but may also have clinical implications, with the cessation of the rebound increase in BDZ plasma level being a useful indicator of the end of intoxication. However, up to our best knowledge, we cannot compare this finding with any literature. Based on this observation, we recommend routine plasma level sampling both prior to and after MARS.

The calculated patient's BDZ elimination half-life of 52 h, based on the last time points without MARS, is well in line with half-lives reported for diazepam, which range from 20 to 56 h. Based on this calculation, elimination of BDZ without MARS would take 16 days; therefore, MARS shortened the recovery of patient for 7 days. This would shorten the stay at ICU, decreased the risks of infections, ventilation-associated lung damage, etc.

However, diazepam is metabolized into the active metabolites nordiazepam and oxazepam, which have substantially longer elimination half-lives of more than 200 h and high protein binding (8, 9). Consequently, the match between calculated BDZ and diazepam half-lives may be explained by the metabolites not being formed at high concentrations and/or complete removal by MARS without substantial accumulation in peripheral tissue. Unfortunately, metabolite levels cannot be discerned by the immunoassay method used.

To the best of our knowledge, our case represents the highest dose of diazepam intoxication ever reported. Greenblatt et al. reported a diazepam poisoning with a 10-fold lower dose, that is, 2,000 mg (10). While they report high concentrations of all metabolites present in early samples, which subsequently declined slowly over the next 2 weeks, the patient had fully recovered and was discharged within 48 h of ingestion. Because of this rapid clinical recovery, Greenblatt et al. propose that recovery from the diazepam overdose was not attributable to rapid elimination of active compounds from the body, but more likely grounded in adaptation or tolerance to the depressant effects. In contrast, the currently reported patient, who took a 10-fold higher dose, remained unconscious until the last MARS session (in the absence of additional sedating medication). Therefore, a rapid spontaneous recovery, as in case of Greenblatt et al., was clearly absent in our case. Additional data are obviously needed to resolve this issue.

Our study has strengths but also limitation: Measurements of plasma BDZ concentration was assessed prior to and following every MARS session, allowing us to show clearly the beneficial effect of MARS. Based on our further calculation (Figure 1) moreover, the patient's recovery with MARS would be 1 week earlier than without MARS, pointing to the important role of MARS in severe BDZ intoxications. However, only total level of BDZ and not the levels of metabolites were estimated, which is the main limitation of our study.

Collectively, based on the observations and favorable outcome of the current case and in absence of official guidelines for severe flumazenil irresponsive BDZ poisoning, we suggest considering the use of MARS elimination treatment with daily sessions together with twice daily monitoring of BZD plasma levels.

## Data Availability Statement

The raw data supporting the conclusions of this article will be made available by the authors, without undue reservation.

## Ethics Statement

Ethical review and approval was not required for the study on human participants in accordance with the local legislation and institutional requirements. The patients/participants provided their written informed consent to participate in this study.

## Author Contributions

AD, AG, AY, and RH made substantial contributions to the conception or design of the manuscript. PV and DV-Y to the acquisition, analysis, and interpretation of the data. RH revised it critically. All authors participated in drafting the manuscript.

## Conflict of Interest

The authors declare that the research was conducted in the absence of any commercial or financial relationships that could be construed as a potential conflict of interest.
